# 3D-printing magnesium–polycaprolactone loaded with melatonin inhibits the development of osteosarcoma by regulating cell-in-cell structures

**DOI:** 10.1186/s12951-021-01012-1

**Published:** 2021-09-04

**Authors:** Weilin Zhang, Wei Zhao, Qin Li, Duoyi Zhao, Junxing Qu, Ziyang Yuan, Zhihong Cheng, Xiaojuan Zhu, Xiuli Zhuang, Zhiyu Zhang

**Affiliations:** 1 Department of Orthopedics, The Fourth Affiliated Hospital of China Medical University, Shenyang, 110032 Liaoning China; 2 Translational Medicine Center, The Fourth Affiliated Hospital of China Medical University, Shenyang, Liaoning China; 3 Key Laboratory of Molecular Epigenetics, Ministry of Education and Institute of Cytology and Genetics, Northeast Normal University, Changchun, Jilin China; 4 Key Laboratory of Polymer Ecomaterials, Changchun Institute of Applied Chemistry Chinese Academy of Sciences, Changchun, Jilin China

**Keywords:** Osteosarcoma, Melatonin, Cell-in-cell, Mg–PCL, Rho/ROCK, cAMP/PKA signaling pathway

## Abstract

Melatonin has been proposed as a potent anticarcinogen presents a short half-life for osteosarcoma (OS). Cell-in-cell (CIC) structures play a role in the development of malignant tumors by changing the tumor cell energy metabolism. This study developed a melatonin-loaded 3D printed magnesium–polycaprolactone (Mg–PCL) scaffold and investigated its effect and molecular mechanism on CIC in OS. Mg–PCL scaffold was prepared by 3D-printing and its characteristic was determined. The effect and molecular mechanism of Mg–PCL scaffold as well as melatonin-loaded Mg–PCL on OS growth and progression were investigated in vivo and in vitro. We found that melatonin receptor 1 (MT1) and CIC expressions were increased in OS tissues and cells. Melatonin treatment inhibit the key CIC pathway, Rho/ROCK, through the cAMP/PKA signaling pathway, interfering with the mitochondrial physiology of OS cells, and thus playing an anti-invasion and anti-metastasis role in OS. The Mg–PCL–MT could significantly inhibit distant organ metastasis of OS in the in vivo model. Our results showed that melatonin-loaded Mg–PCL scaffolds inhibited the proliferation, invasion and metastasis of OS cells through the CIC pathway. The Mg–PCL–MT could be a potential therapeutics for OS.

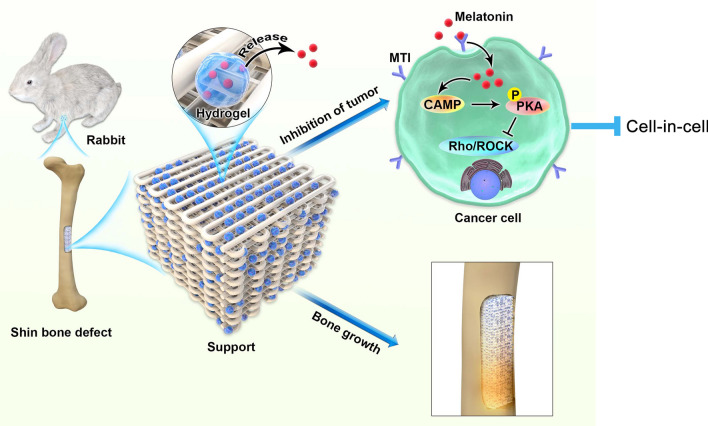

## Introduction

Osteosarcoma (OS), the most common type of malignant bone tumor [1], originates from the abnormal transformation of mesenchymal cells, and exhibits osteoblastic differentiation with the production of malignant bone-like substances [2]. OS is the most common bone cancer in adolescents and is one of the leading causes of death in children [3]. The incidence of OS is sex-related, with a higher rate in male population [4], and mainly occurs in the metaphysis of long bones. Although complete radical excision of the extensive diseased extremity and en bloc resection of the tumor has been widely applied to treat OS, the mortality rate still remains high due to the high potential of metastasis [5]. Besides, implants need to be implanted when tumor resection causes bone defect and therefore, bone regeneration materials are attracted much attention.

Magnesium is the second most abundant caption in body and plays an essential role in metabolic function [6, 7]. More than half of the total Mg is distributed in bones [8]. It is reported that Mg ion is essential for new bone formation and could induce increase in osteogenic activity, as well as maintaining vascular function [9, 10]. The Mg and its alloys are non-toxic, of excellent biodegradability, suitable mechanical compatibility, desirable osteoconductivity, biocompatibility and osseointegrative properties [11–13]. Therefore, they were considered as an ideal candidate for using as temporary, degradable orthopedic implants. However, Mg alloys are susceptible to corrosion in a biological environment [14], which is the main obstacle to their clinical use. Polycaprolactone (PCL) is a kind of synthetic polymer with a low degradation rate and desirable mechanical properties [15, 16]. PCL has been utilized as a surface coating on Mg alloys in order to improve the corrosion resistance. Studies have shown that PCL has a slower degradation rate and exhibits biocompatibility on Mg [17, 18]. Mg–PCL composite offers an ideal candidate for bone tissue engineering with lower corrosion resistance and good biocompatibility.

Several cancer drug-loaded bone regeneration materials have been developed. Melatonin, a hormone secreted by the pineal gland [19], exerts physiological effects on the circadian rhythm, mood, sleep and aging [20]. Recent evidence has suggested that melatonin also has an impact on the treatment of many kinds of cancer, including OS, colon cancer, breast cancer, prostate cancer, and gastric cancer [21, 22]. It has been reported that melatonin could regulate bone formation, growth and differentiation as well as exert anti-osteoporosis effects [23, 24]. Because melatonin presents variable, low bioavailability and a short half-life, its clinical application is significantly hindered. Besides, systemic administration requires a large amount of melatonin, which might lead to drug side effects. Therefore, drug delivery system, such as polymeric nanoparticulated systems might provide promising alternatives [25].

Previous studies have reported that melatonin can affect intracellular cAMP level by acting on melatonin receptor 1 (MT1) [26], and it has been reported that cAMP regulates the formation of Cell-in-cell (CIC) formation through the Rho/ROCK pathway [27]. CIC structures refer to the phenomenon whereby one or more living cells are internalized into adjacent living cells [28, 29]. The CIC process is caused by the loss of matrix adhesion, leading to invasion of one or more living cells into another. The discovery of CIC can be dated back to more than 100 years ago; however, its research only attracted great interest in recent years because of its potential pathophysiological significance [30]. CIC formation between tumor cells has been reported to alter energy metabolism, which closely related to the occurrence and development of tumors [31, 32]. Considerable evidence has suggested that the CIC phenomenon could promote tumor metastasis, except for pancreatic cancer and influence cancer prognosis [33, 34]. However, the effect of melatonin on CIC formation in OS has not been fully elucidated.

Three-dimensional (3D) printing technology is a precise, fast and controllable fabrication technology which fits the aim of personalized treatment nowadays [35]. 3D printed materials have been utilized into many fields [36]. In this study, we loaded melatonin in a 3D printing Mg–PCL scaffold and investigated its effects and molecular mechanism on inhibiting OS growth and metastasis. We hypothesized that the melatonin-loaded Mg–PCL implants affects CIC formation through the cAMP/Rho/ROCK pathway and thereby inhibiting OS growth. Our results might provide a new therapeutic strategy for treating OS.

## Methods

### Patients

From February 2015 to December 2018, 30 fresh OS patient tissue samples were collected from the Department of Orthopedics, the Fourth Affiliated Hospital of China Medical University. After collection, the samples were immediately stored in liquid nitrogen at − 80 °C for future use. Detailed information of each sample is shown in Table 1. All procedures performed in studies involving human participants were in accordance with the ethical standards of the institutional and national research committee and with the 1964 Helsinki declaration and its later amendments or comparable ethical standards. Written informed consent has been received from all included patients and the research protocol has been approved by the Fourth Affiliated Hospital of China Medical University Ethics Committee (No. EC-2018-KS-058).

**Table 1:** Clinicopathological features of OA patients (n = 30)

Characteristics	Number of OA patients	Percent (%)
Gender		
Male	15	50
Female	15	50
Age, years		
≤ 25	17	56.7
> 25	13	43.3
Tumor site		
Tibia	16	53.3
Femur	6	20
Other	8	26.7
TNM staging		
I−II	10	33.3
III−IV	20	66.7
Distant metastasis		
Yes	12	40
No	18	60

### Reagents

Pentanoic acid (HY-N6056), ROCK inhibitor-2 (HY-119937), S26131 (HY-122136) and 7-Desmethyl-agomelatine (HY-133113) were obtained from Med Chem Express (MCE, New Jersey, USA). SunP Gel G1 was purchased from SunP Biotech (Beijing, China). Bovine serum albumin (BSA) was purchased from Sigma-Aldrich. Anti-E-cadherin (ab1416), anti-MTNR1A (ab203038), anti-MTNR1B (ab203346), anti-tubulin (ab210797), Alexa 488 (ab150077) and Alexa 647 (ab150115) were purchased from Abcam (Cambridge, UK). Anti-COX4I1 (11242-1-AP) and anti-ATPB (17247-1-AP) were purchased from Proteintech (Hubei, China). Anti-TOM20 (#42406), anti-PGC1α (#2178), anti-NRF1 (#46743), and anti-TFAM (#8076) were obtained from Cell Signaling Technology (Massachusetts, USA). Anti-glyceraldehyde-3-phosphate dehydrogenase (GAPDH, TA309157) and all secondary antibodies were purchased from Zsbio (Beijing, China).

### 3D-printing of Mg–PCL scaffolds

Different ratios (W/W) of Mg powder (1%, 2.5%, 5% and 10%) and PCL (MW: 80,000 Da) were weighed and mixed at high temperature with an internal mixer for 10 min. The temperature was set at 120 °C and the rotating speed was set at 20 rpm. After mixing, the material was cut into small particles. Scaffolds were printed by the 3D printer (BioMaker, Ubbiotech, China) using SolidWorks 2017 software and Simplify 3D software. Printing parameters were set as follows: printing temperature was 120 °C, filling rate was 50%, and printing speed was 6 mm/s.

### Melatonin loading on the scaffold

Melatonin and SunP Gel G1 were added into 10 ml Phosphate Buffer Saline (PBS) with a mass ratio of 1:4, 1:3, 1:2, 1:1 and 2:1. The mixture was stirred at 650 r/min for 2 h in a water bath at 40 °C to completely dissolve. After being cooled to room temperature (23–25 °C), the MT-loaded gel was obtained. MT-loaded SunP Gel G1 gel (300 µl) was injected into the gap of the 3D Mg–PCL scaffold. The scaffold was placed at room temperature (23–25 °C) to achieve liquid-solid conversion to obtain MT-loaded Mg–PCL scaffold.

### Determination of characterisics of Mg–PCL scaffold

Chemical composition of the scaffold was determined by Fourier transform infrared spectroscopy (FTIR, Perkin Elmer, FTIR-2000). For thermogravimetric analysis (TGA), samples were heated at 10 °C/min in open aluminium pans with a Discovery TGA (TA instruments, Waters, LLC, USA). Nitrogen was used as a purge gas with a flow rate of 25 ml/min. Data collection and analysis were performed using TA Instruments Trios software and % mass loss and/or onset temperature were calculated. The wide-angle X-ray diffraction (XRD) pattern of chitin powders and the dried sheets were recorded on an XRD instrument (D8ADVANCE, BRUKER, Germany) with Cu–K radiation (λ = 0.154 nm). The XRD data were collected from 2θ = 5 to 35° at a scanning rate of 2° min^−1^. The mechanical properties of the scaffold were measured using a universal mechanical testing machine (Instron 1121, UK). Scanning electron microscopy (SEM) Surface and cross-section images of the filaments were taken with an SEM (JSM-840 A Scanning Microscope, JEOL GmbH, Eching, Germany).

### In vitro immersion test

The samples were weighted and recorded. Then, the samples were placed into clean 15 ml tubes. Hank’s solution was then added to the tipped plastic bottle at a ratio of the surface area of the sample of 1 cm^2^: 25 ml. The soaking bottle was placed in a WE-1 type water bath thermostat at 37 °C. pH was recorded every day. After 30 days, the samples were dried with a hair dryer. The surfaces were photographed under SEM. Afterwards, the samples were ultrasonically cleaned by chromic acid solution (200 g/l CrO_3_), distilled water and ethyl alcohol for 10 min. The samples were weighted again after being dried by a hair dryer. The corrosion rate was calculated using the follow formula:

$${\text{Corrosion rate}} = \left( {{\text{K}} \times {\text{W}}} \right)/\left( {{\text{A}} \times {\text{T}} \times {\text{D}}} \right)$$

where K equals 8.76 × 10^4^; W is the weight difference before and after soaking (g); A is the surface area of the sample exposed to Hank’s solution (cm^2^); T is the soak period (h) and D is the density of samples.

### Determination of loading efficiency (LE) and loading capacity (LC)

Melatonin solution was prepared using absolute ethanol and chloroform (V: V = 9:1) as solvent. The wavelength of the maximum absorption peak was measured at the wavelength range of 200–350 nm using an ultraviolet-visible spectrophotometer (Mettler Toledo, Switzerland). Standard curve was draw by linear regression fitting of the absorbance with the mass concentration of the melatonin solution at the wavelength of the maximum absorption peak (278 nm). Freeze-dried melatonin-loaded SunP Gel G1 gel (50 mg) was dissolved in PBS (10 ml). After being centrifugation at 12,000 rpm for 5 min, 0.5 ml supernatant was diluted in PBS in a 25 ml volumetric flack. The absorbance value of the solution was measured at 278 nm. The mass of melatonin was calculated from the standard curve and the LE and LC was calculated by following formula:

$${\text{LE}} = {{\text{m}}_{\text{a}}}/{{\text{m}}_{\text{b}}} \times 100\%$$

$${\text{LC}} = {{\text{m}}_{\text{a}}}/{{\text{m}}_{\text{c}}} \times 100\%$$

where m_a_ means the mass of melatonin in melatonin-loaded SunP Gel G1 gel, m_b_ represents total mass of melatonin and m_c_ is the mass of SunP Gel G1 in melatonin-loaded SunP Gel G1 gel.

### In vitro release of melatonin from melatonin-loaded scaffolds

Standard curve of melatonin was first draw using PBS (pH = 7.4) as solvent under the wavelength of the maximum absorption peak (278 nm). Melatonin-loaded SunP Gel G1 gel (100 mg) was dissolved in PBS and placed in a dialysis bag. The dialysis bag was then placed in a beaker containing the same pH PBS buffer and was vibrated horizontally in a constant temperature shaker (37 °C ± 0.5 °C) with a vibration frequency of 50 times/min. The release liquid (5 ml) was replaced with same amounts of fresh PBS at selected time intervals. The absorbance value of the solution was measured at 278 nm and the drug release rate was calculated as follow:

$${\text{R}}( \%) = \frac{{{\rho _n}V + {V_i} \mathop \sum \nolimits_t^{n - i} {\rho _i}}}{{{M_D}}} \times 100$$

where R means the cumulative drug release rate (%); n means the number of sampling. ${\rho }_{n}$ represents the mass concentration (g/l) of the drug in the nth release. V represents the total volume of the release liquid. ${\rho }_{i}$ represents the mass concentration (g/l) of the drug in the ith release, V_i_ represents the volume of the release liquid at the ith sampling and M_D_ means the mass of drug loaded (g).

### Pharmacokinetics of melatonin

The chromatographic conditions for the high-performance liquid chromatography (HPLC) analysis of melatonin were as follows: Hypersil ODS2 column (150 mm × 4.6 mm, 5 μm); mobile phase containing 20 mmol l^−1^ NaAc-HAC buffer solution (pH = 3.4)/methanol (65:35 v/v); flow rate of 1.0 ml min^−1^; excitation (Ex) and emission (Em) detection wavelengths of 285 mm and 345 mm, respectively; and column temperature of 35 °C.

For sample treatment, 200 µl of mice plasma was added into 1.2 ml of ethyl acetate for extraction, and vortex mixed for 2 min. Following centrifugation for 10 min (150,000 R min^−1^, 4 °C), 1 ml of the ethyl acetate layer (upper layer) was transferred into another centrifuge tube, and dried with nitrogen. The residue was then re-dissolved in 100 µl of mobile phase, vortex mixed for 30 s, and centrifuged for 10 min (150,000 R min^−1^), to allow 20 µl of the supernatant to be drawn for injection.

### Animal experiment of mice for OS growth

Female Balb/c nude mice (n = 6, 8 weeks old) were purchased from the Experimental Animal Center of Jilin University. The mice were bred under temperature-controlled conditions at 22.6 ± 2 °C with a 12-h day/light cycle. All applicable institutional and national guidelines for the care and use of animals were followed and the animal experiments were approved by the Animal Care and Use Committee of China Medical University. The heterograft mouse model was established by subcutaneous injection of 2 × 10^8^ human OS (U2OS) cells suspended in 0.2 ml medium into the root of the right thigh.

When the tumors grew to about 80 mm^3^, the mice were randomly divided into 8 groups: (1) control group: mice received intraperitoneal treatment of PBS (vehicle); (2) MT group: mice received intraperitoneal injection of melatonin (40 mg kg^−1^ day^−1^); (3) MT + ROCK activator group: mice received intraperitoneal injection of melatonin (40 mg kg^−1^ day^−1^) and ROCK activator (10 mg kg^−1^day^−1^), respectively; (4) MT + ROCK inhibitor group: mice received intraperitoneal injection of melatonin (40 mg kg^−1^ day^−1^) and ROCK inhibitor (10 mg kg^−1^ day^−1^), respectively; (5) Mg–PCL group: Mg–PCL scaffold (10 mm * 2.5mm * 2.5 mm) was subcutaneous implanted into the root of the right thigh, next to the tumor; (6) Mg–PCL + MT group: melatonin was loaded into Mg–PCL scaffold with SunP Gel G1 and was subcutaneous implanted into the root of the right thigh, next to the tumor; (7) Mg–PCL + MT + ROCK activator group: MT-loaded Mg–PCL scaffold was subcutaneous implanted into the root of the right thigh, next to the tumor; mice received intraperitoneal injection of ROCK activator (10 mg kg^−1^ day^−1^); (8) Mg–PCL + MT + ROCK inhibitor group: MT-loaded Mg–PCL scaffold was subcutaneous implanted into the root of the right thigh, next to the tumor; mice received intraperitoneal injection of ROCK inhibitor (10 mg kg^−1^ day^−1^). All treatments sustained for 27 days.

The mice were sacrificed. Tumors and main organs (heart, liver, spleen, lung and kidney) were collected, weighed, fixed overnight in 4% paraformaldehyde, and then embedded in paraffin. The paraffin-embedded samples were cut into a thickness of 5 μm and used for the staining of hematoxylin and eosin (HE). Histological alterations were evaluated using a fluorescence microscope (Nikon Eclipse Ti, Optical Apparatus Co., Ardmore, USA) and a confocal microscope (Carl Zeiss, LSM 780).

### Animal experiment of rabbits for OS metastasis

New Zealand white rabbits (2-month old) weighing 2.0–3.0 kg were purchased from the animal experiment center of China Medical University. VX2 sarcoma cell line was purchased from Shanghai Yubo Biotechnology Co., Ltd and the tumor was passaged by continuous passage of tumor-bearing rabbits. The tumor was excised from the lower limbs of the sacrificed tumor-bearing rabbits. After removed the blood vessels and fibrous tissue around the tumor tissue with ophthalmic scissors, the tumor was cut into small pieces and immersed in normal saline for use.

The rabbits were routinely anesthetized. The plane of the front edge of the proximal tibia of the rabbit was fully exposed. A 1.5 cm incision was made longitudinally on the tibia. The fascia and periosteum were peeled off to expose the tibial cortex. A 1 mm diameter hole was made in the cortex using a dental drill. Two to three pieces of tumor tissue with a volume of about 1 mm^3^ were inoculated into the medullary cavity. The orifice and the surrounding 1 cm area were sealed with bone wax. The fascia and skin were sutured sequentially. One month after the tumor was implanted and an incision was made layer by layer along the longitudinal axis of the tumor to expose the tibia, to verify the modeling of the OS model.

The OS model animals were divided into control group (without any implant), a titanium–PCL (Ti–PCL) group, a Mg–PCL implant group (Mg–PCL), and a Mg–PCL carrying melatonin group (Mg–PCL–MT). The bone tumor was completely removed. A hole was drilled at the front edge of the tibial plateau, and the implant was inserted into the bone marrow cavity. Two months after the operation, the experimental animals were sacrificed, the rabbit tibia was completely removed for MicroCT detection, and the rabbit organs (heart, liver, spleen, lung, and kidney) were taken for HE staining.

### HE staining

Tissues were fixed in 4% paraformaldehyde. After fixation, the samples were decalcified in 10% EDTA for 21 days before paraffin embedding and then sliced microtomically into a systematic series of 16 μm sections. Standard HE staining was performed and photographed. The average number of deposits per section was calculated using Image-Pro Plus 6.0 (Media Cybernetics, USA).

### Immumohistochemical staining

Tumor sections were treated with 0.6% H_2_O_2_ in PBS for 20 min and then washed with Tris-buffered saline (TBS) for three times. After that, sections were blocked in 0.5% Triton-x-100 in TBS and Triton X-100/3% horse serum (TBS-TS) for 60 min at room temperature (23–25 °C), and incubated with primary antibody ki-67 at 1:400 in TBS-TS at 4 °C for 48 h. Then after four times of washing with TBS, sections were incubated with secondary goat-rabbit IgG antibodies (1:1000, Bioworld) at room temperature (23–25 °C) for 2 h. After three times of washing with TBS, DAB solution was added to incubate for 5 min. Images were obtained with a DP72 digital camera (Olympus, Tokyo, Japan) and DP2-BSW microscope digital camera software (Olympus).

### Tunnel assay

Tunnel assay was performed according to the instructions of commercially available kit (Beyotime C1086). Tumor sections were fixed with h 4% paraformaldehyde at room temperature (23–25 °C) for 15 min. Fixed cells were permeabilized with 0.2% Triton X-100 and then blocked with 5% BSA in PBS. After these basic operations, sections were treated with tunnel staining according to the kit’s specification. Images were obtained with a confocal laser scanning microscope (Olympus, Tokyo, Japan).

### MicroCT scan

The tibias of rabbits or mice were aseptically removed and assessed by MicroCT scan as described previously [37]. The bone mineral density (BMD), trabecular number (Tb.N), trabecular bone volume per tissue volume (BV/TV) and trabecular thickness (Tb.Th) were determined.

### Cell culture

The human fetal osteoblastic cell line, hFOB1.19, and OS cell line, U2OS, MG63, HOS and 143B, were purchased from the Cell Bank of the Chinese Academy of Sciences (Shanghai, China). The cells were suspended in a 1:1 mixture of Ham’s F12 medium and Dulbecco’s Modified Eagle’s Medium (DMEM/F12) (HyClone, Utah, USA), 10% fetal bovine serum (HyClone) and 0.3 g/l G418 (Sigma-Aldrich, Darmstadt, Germany), and maintained in a humidified 5% CO_2_ atmosphere at 33.5 °C. The medium was changed every 2 days, and the cells were passaged using Trypsin-EDTA (HyClone). The cells were plated at 10^4^ cells/cm^2^ for subsequent experiments.

U2OS cells were divided into 8 groups: (1) Control group: without any treatment; (2) MT group: U2OS cells were added with 1 mM melatonin; (3) MT + ROCK activator group: U2OS cells were added with 1mM melatonin and 50 µM ROCK activator (pentanoic acid); (4) MT + ROCK inhibitor group: U2OS cells were added with 1mM melatonin and 50 µM ROCK inhibitor (ROCK inhibitor-2); (5) Mg–PCL group: U2OS cells were co-cultured with Mg–PCL scaffold; (6) Mg–PCL + MT group: U2OS cells were co-cultured with Mg–PCL scaffold loaded with melatonin; (7) Mg–PCL + MT + ROCK activator group: U2OS cells were co-cultured with Mg–PCL scaffold loaded with melatonin and added with 50 µM ROCK activator (pentanoic acid); and (8) Mg–PCL + MT + ROCK activator group: U2OS cells were co-cultured with Mg–PCL scaffold loaded with melatonin and added with 50 µM ROCK inhibitor (ROCK inhibitor-2).

### Transmission electron microscopy (TEM)

The cells were scrapped after treatment, collected by centrifugation, washed with PBS and fixed with 5% glutaraldehyde. After the cells were dehydrated, embedded, sectioned, stained, the mitophagosomes were observed by TEM. The quantity of CIC structures was calculated in 10 fields a view.

### Western blot analysis

Cells or tissues were washed with ice-cold PBS and resuspended in lysis buffer (50 mM Tris–HCl, 150mM NaCl, 1% NP-40, 0.5% sodium deoxycholate, 0.1% sodium dodecyl sulfate) containing protease and phosphorylase inhibitor cocktails. After centrifugation at 12,000*g* for 30 min at 4 °C, the supernatant containing total protein was collected and quantified using the BCA protein concentration assay kit (Boster, Hubei, China). The samples were separated by SDS-PAGE and then transferred to polyvinylidene difluoride (PVDF) membranes (Millipore, Massachusetts, USA) (210 mA, between 30 and 120 min according to the molecular mass of the protein). After blocking for 2 h with 5% non-fat milk, the PVDF membranes were incubated with the appropriate primary antibodies (1:500 or 1:1000 dilution) at 4 °C overnight. The blots were then incubated with anti-mouse or anti-rabbit IgG conjugated to horseradish peroxidase (1:5000 dilution) for 1 h at room temperature (23–25 °C). Immunoreactive bands were visualized by the EC3 imaging system (UVP Inc., California, USA), and quantified with the ImageJ software (NIH, USA). The ratios between the proteins of interest and loading controls of the same sample were calculated as relative content and expressed graphically. The results were averaged from three independent experiments.

### Immunofluorescence

Frozen sections of human OS tissue were stained with E-cadherin, MT1 and MT2 primary antibodies, followed by Alexa 488-conjugated goat anti-rabbit (Abcam) and Alexa 647-conjugated goat anti-mouse (Abcam) secondary antibodies. Image-Pro Plus 6.0 was used for the quantitative analysis of protein expression, and a mean optical density value [integrated optical density (IOD) divided by the relevant area] was calculated for each visual field.

After treatment, the cells were fixed with 4% paraformaldehyde at room temperature (23–25 °C) for 15 min. After PBS washing, cells were permeabilized with 0.2% Triton X-100 for 5 min. Sections were incubated in a blocking buffer containing 5% BSA for 30 min at room temperature (23–25 °C), followed by incubation with primary antibodies overnight at 4 °C. Secondary antibodies labeled with fluorescein (1:500, Abcam) were applied for 120 min. After incubating with 0.1% DAPI for 10 min and another wash with PBS, images were captured on a wide field fluorescent microscope (Olympus, Japan). Each experiment was repeated three times (total, n = 90 cells) and the fluorescence intensities were quantified with the ImageJ software. Cells were grown in confocal dishes (Nest, California, USA) and incubated with Mitotracker Red CMXRos (M7512, Invitrogen) for 30 min to mark the mitochondria according to the manufacturer’s protocol. The cells were then washed with ice-cold PBS, and analyzed under a confocal laser scanning microscope (Olympus, Tokyo, Japan). Data were averaged from three independent experiments (total, n = 90 cells). Image-Pro Plus 6.0 (Media Cybernetics) was used for quantifying mitochondria.

### Cell invasion and migration assays

To examine the effects of melatonin and CIC formation on the invasiveness of U2OS cells in vitro, we employed a modified Boyden chamber invasion assay with Matrigel coating. After treatment, the cells were seeded into the upper section of the Boyden chamber (Neuro Probe, Cabin John, MD, USA) at densities of 2.0 × 10^5^/ml, and then incubated for 24 h at 37 °C. Finally, the cells were counted under a light microscope. Cell migration was measured by a cell invasion assay without Matrigel coating.

### Wound-healing assay

The U2OS cells were plated at 8.5 × 10^5^/well on 6-well plates for 48 h and were wounded by scratching with a pipette tip. They were subsequently incubated with DMEM containing 0.5% FBS. After repeated treatment at 0, 12, 24 and 48 h, the cells were photographed using a phase contrast microscope (Olympus, Japan).

### Adenosine triphosphate (ATP) and oxygen consumption measurement

Mitochondria were separated by the Cell Mitochondria Isolation Kit (Beyotime, Shanghai, China). Mitochondrial ATP levels were measured by the ATP Assay Kit (Sigma, MAK 190); while endogenous basal oxygen consumption was measured using Clark-type electrodes of the Oxygen Consumption Rate Assay Kit (Cayman, 600800). All procedures were done according to the manufacturer’s protocol.

### DNA isolation and mitochondrial DNA copy number quantification

Genomic DNA was isolated using the EasyPrep Genomic DNA Extraction Kit (TOOLS) following the manufacturer’s instructions. Mitochondrial DNA (mtDNA) copy number was quantified in 10 µl 2X SYBR Green PCR Master Mix (Roche) containing 5 µM forward and reverse primers, with approximately 10 ng DNA. mtDNA levels were assessed using primers against the mitochondrial gene, ND1, with telomerase reverse transcriptase (TERT) serving as a loading control. Gene expression quantification was based on the comparative cycle threshold (CT) (2^−△△CT^) method. The primer sequences were as follows: ND1: forward, 5′-ACCAT TTGCA GACGC CATAA-3′; reverse, 5′-TAAAT TGTTT GGGCT ACGG-3′; TERT: forward, 5′-CTAGC TCATG TGTCA AGACC CTCT-3′; reverse, 5′-GCCAG CACGT TTCTC TCGTT-3′.

### Statistical analysis

The quantitative variables are expressed as mean ± standard deviation (SD). All data were analyzed using the GraphPad Prism 6.02 software program. Statistical significance between the two groups was analyzed by the Student’s t-test. The one-way analysis of variance (ANOVA) was used to compare multiple groups. A P value of < 0.05 was considered statistically significant.

## Results

### Determination of the characteristics of Mg–PCL scaffold

**Fig. 1:**
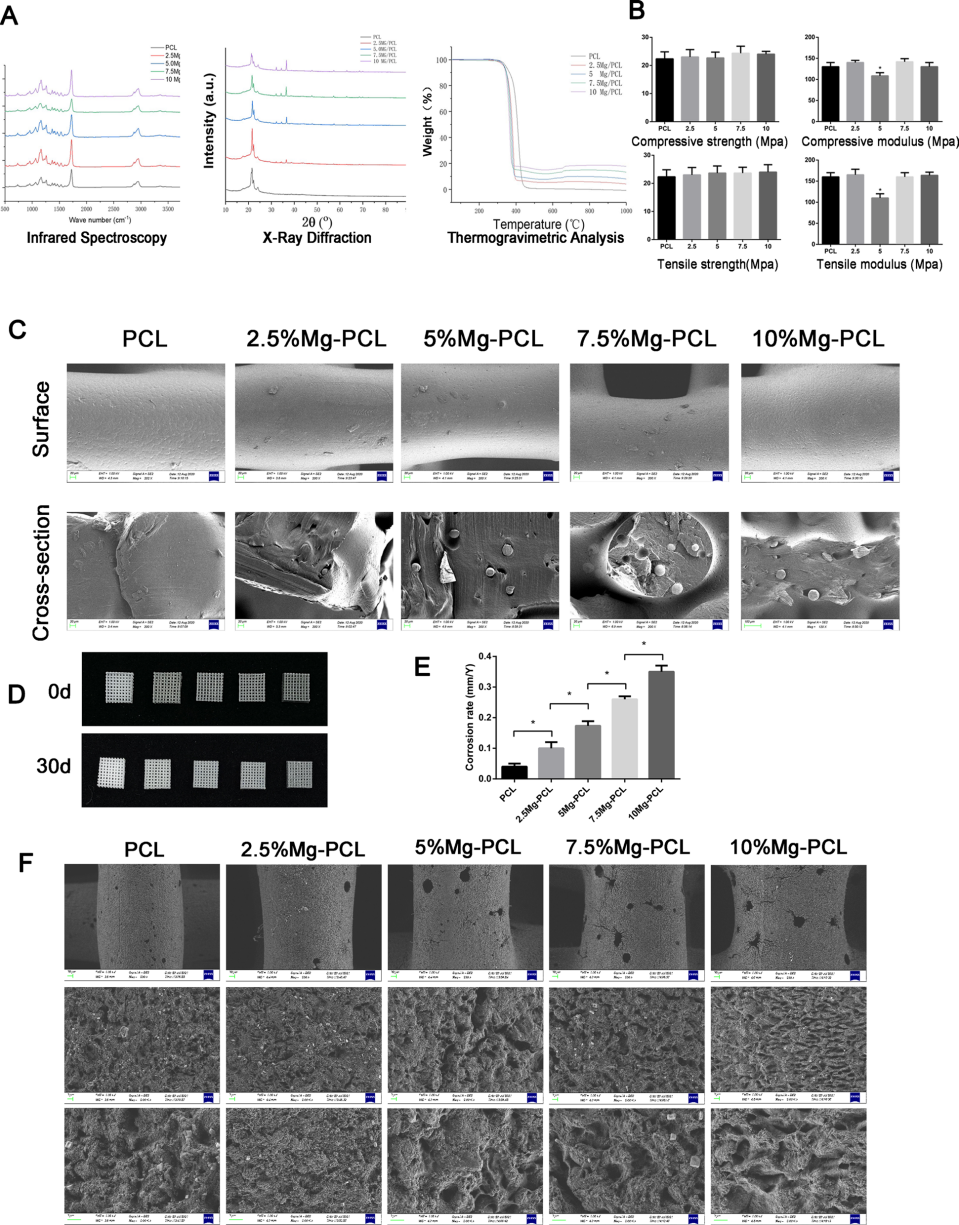
Characteristics of Mg–PCL scafford. **A** Infrared spectroscopy, X-Ray diffraction and thermogravimetric analyses of Mg–PCL. **B** Compressive and tensile characteristics of Mg–PCL. * P < 0.05 compared with PCL group. **C** The surface and cross-section images of the Mg–PCL scaffold with different content of Mg under scanning electron microscopy. **D** In vitro immersion test of Mg–PCL scaffold with different content of Mg at 0-day and 30-day. **E** Corrosion rate of Mg–PCL scaffold with different content of Mg at 0-day and 30-day. * P < 0.05. **F** The surface morphology of Mg–PCL scaffold with different content of Mg was observed by SEM at 30-day

Mg–PCL composite offers an ideal candidate for bone tissue engineering with lower corrosion resistance and good biocompatibility. We first prepared an Mg–PCL scaffold by 3D-printing. The characteristics of Mg–PCL scaffold were determined. The infrared spectroscopy of PCL was not significantly changed after mixing with Mg. TGA analysis suggested that PCL was sublimated at 412 °C and the sublimation temperature decreased with the increase of Mg content (Fig. 1A). Mechanical properties of the scaffold were not significant different among different groups, except that the compressive modulus was significantly decreased at 5% Mg–PCL (Fig. 1B). SEM showed that the Mg content was not much at the surface but distributed primarily in the interior of scaffold (Fig. 1C). In vitro immersion test indicated that the mineralization thickness and corrosion rate of scaffold was gradually increased along with the increasing of Mg content (Fig. 1D–F). Taking physicochemical properties and immersion test into account, we selected the 5% Mg–PCL for further experiment.

### Determination of the effects of Mg–PCL scaffold on the tumor metastasis in vivo

**Fig. 2:**
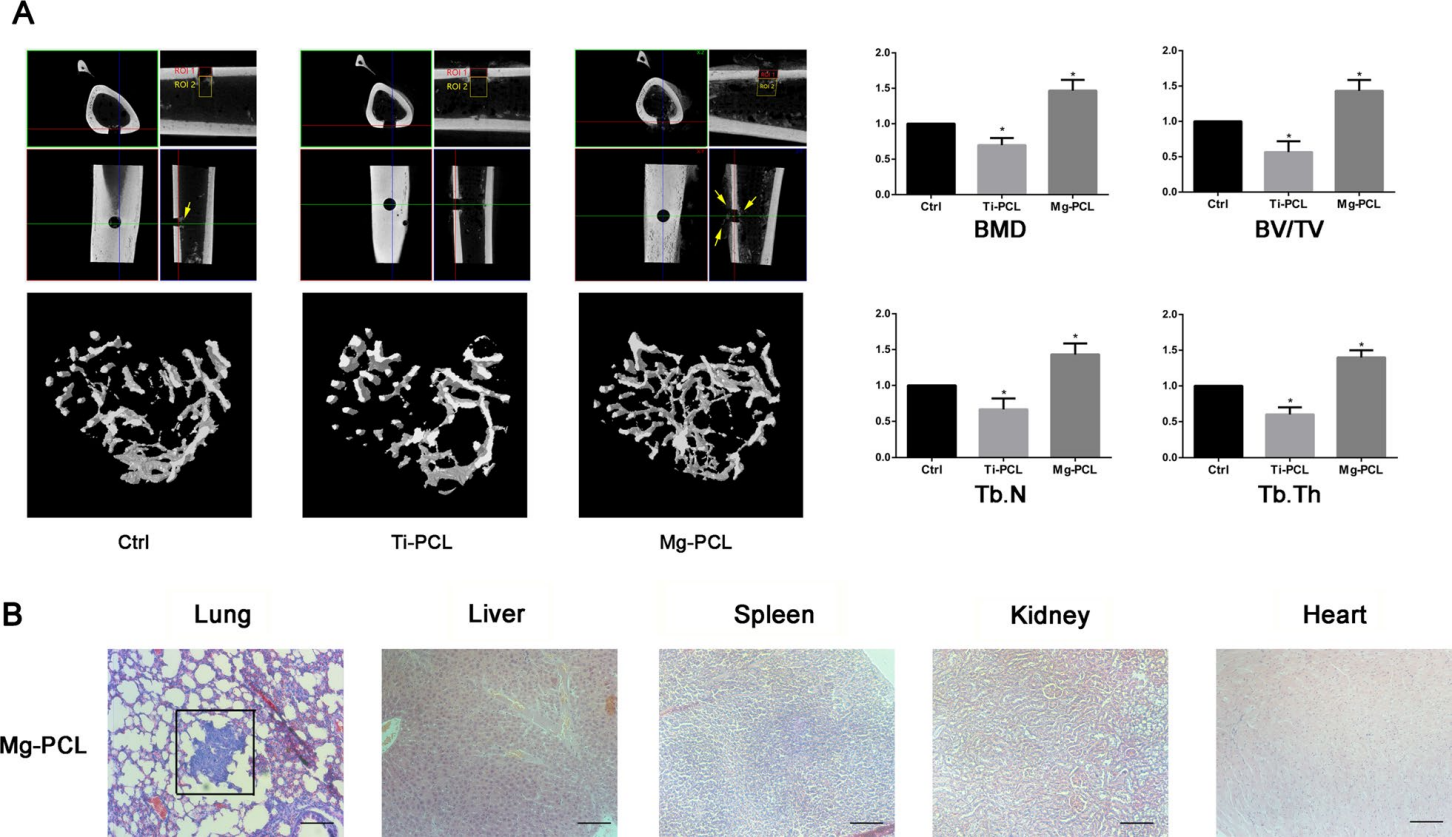
In vivo inhibition effects on U2OS tumor metastasis. **A** MicroCT scan of tibia of rabbit from Control, Ti–PCL and Mg–PCL groups, respectively. BMD, BV/TV, Tb.N and Tb.Th were determined and compared. ROI, Region of interest; BMD, bone mineral density; Tb.N, trabecular number; BV/TV, trabecular bone volume per tissue volume; Tb.Th, trabecular thickness. The arrows indicated the newly formed callus. *P < 0.05 vs. control. **B** HE staining of different organs after 2 months

Further, we established an in vivo OS model in rabbit and inserted the Ti–PCL and Mg–PCL implants into the front edge of the tibial plateau. MicroCT scan results of rabbit tibia showed that the formation of callus in the Mg–PCL scaffold group was increased and the static bone microstructure index was the highest in the three groups, while the traditional Ti–PCL stent was the lowest in the three groups (Fig. 2A). HE staining demonstrated that lung metastasis occurred 2 months after surgery in the Mg–PCL scaffold stent group (Fig. 2B). These data indicated the Mg–PCL scaffold could promote bone growth and might be an ideal candidate for bone tissue engineering; however, it can’t inhibit tumor metastasis.

### Assessment of CIC in tumor tissues of OS patients

**Fig. 3:**
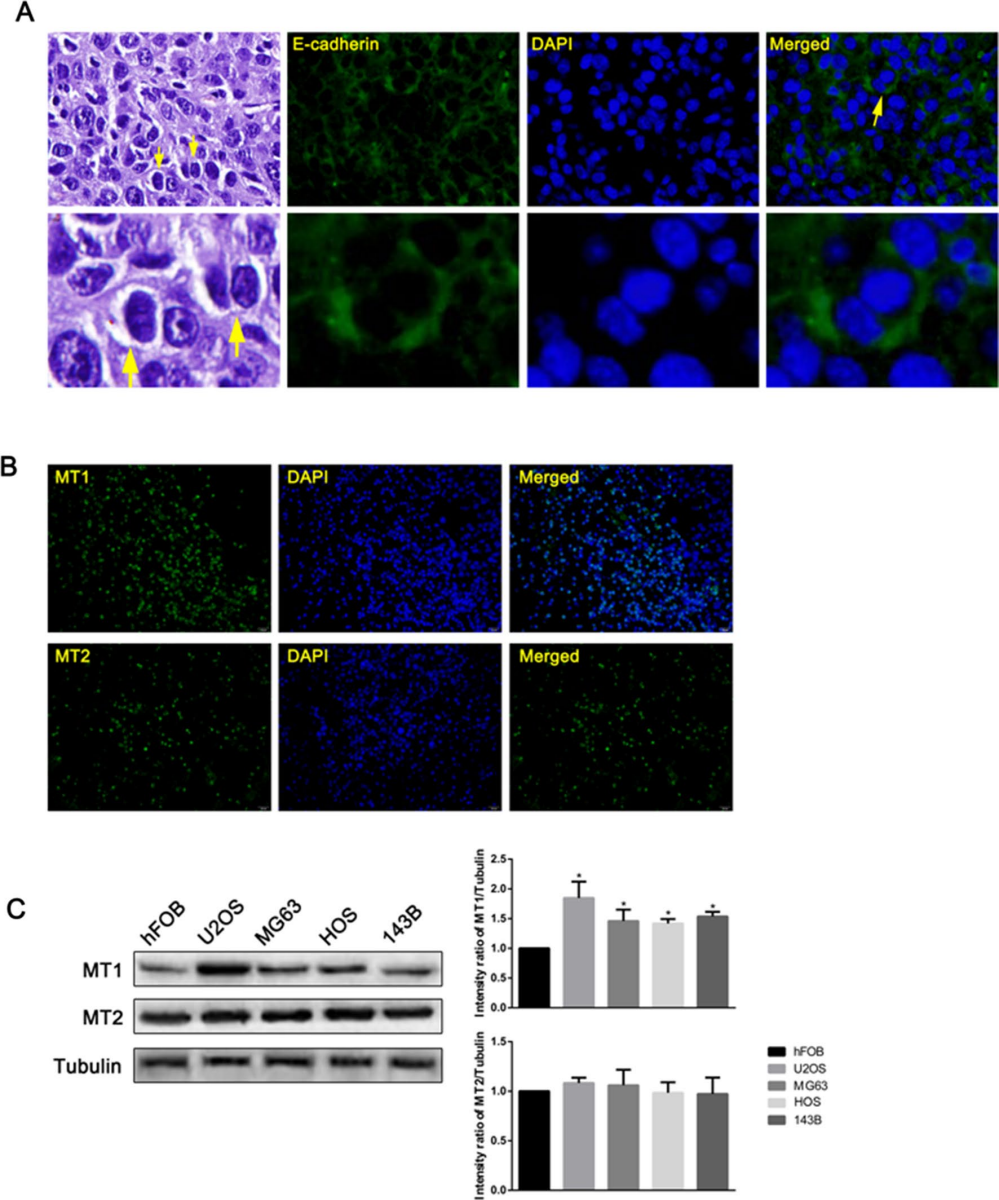
Cell-in-cell (CIC) structure and expression of melatonin receptor in OS patients and OS cells. **A** HE and immunofluorescence of CIC in tumor tissue of OS patients. **B** Immunofluorescence showing MT1 and MT2 expressions in human OS tissues. **C** Western blot shows MT1 and MT2 expression in hFOB, U2OS, MG63, HOS and 143B cells. Values represent the mean ± SD of three independent experiments. *P < 0.05 vs. control

It is reported that CIC phenomenon may promote tumor progression in many types of epithelial tumors [34]. However, its role in OS has not been reported yet. Tumor tissue samples from 30 OS patients (20 fields per sample) were stained with HE and fluorescent-labeled E-cadherin antibody. As shown in Fig. 3A, both HE and immunofluorescence detected CIC phenomenon. These results indicated that CIC occurred in OS tumor tissues, and mainly by tumor cells swallowing other tumor cells.

### Melatonin inhibits OS development through acting on MT1

Melatonin was reported to be an anti-tumor agent for OS by acting on MT1 and MT2 [21, 22]. Immunofluorescence analysis showed that both MT1 and MT2 were expressed in the OS tissues (Fig. 3B). MT1 and MT2 protein expressions in different OS cell lines—U2OS, MG63, HOS and 143B were tested through Western blotting with human osteoblasts hFOB1.19 as the control. The expressions of MT1 in all OS tissues and OS cells were significantly higher, while MT2 expression showed no marked difference compared with the control group (Fig. 3C). Among the OS cell lines, the difference in MT1 protein in U2OS cells was the most obvious. Consequently, the U2OS cell line was selected for the rest of the experiment. We also speculated that effects of melatonin on OS cells might be achieved through MT1.

### Effect of melatonin on CIC formation and OS cell invasion and migration in vitro

**Fig. 4:**
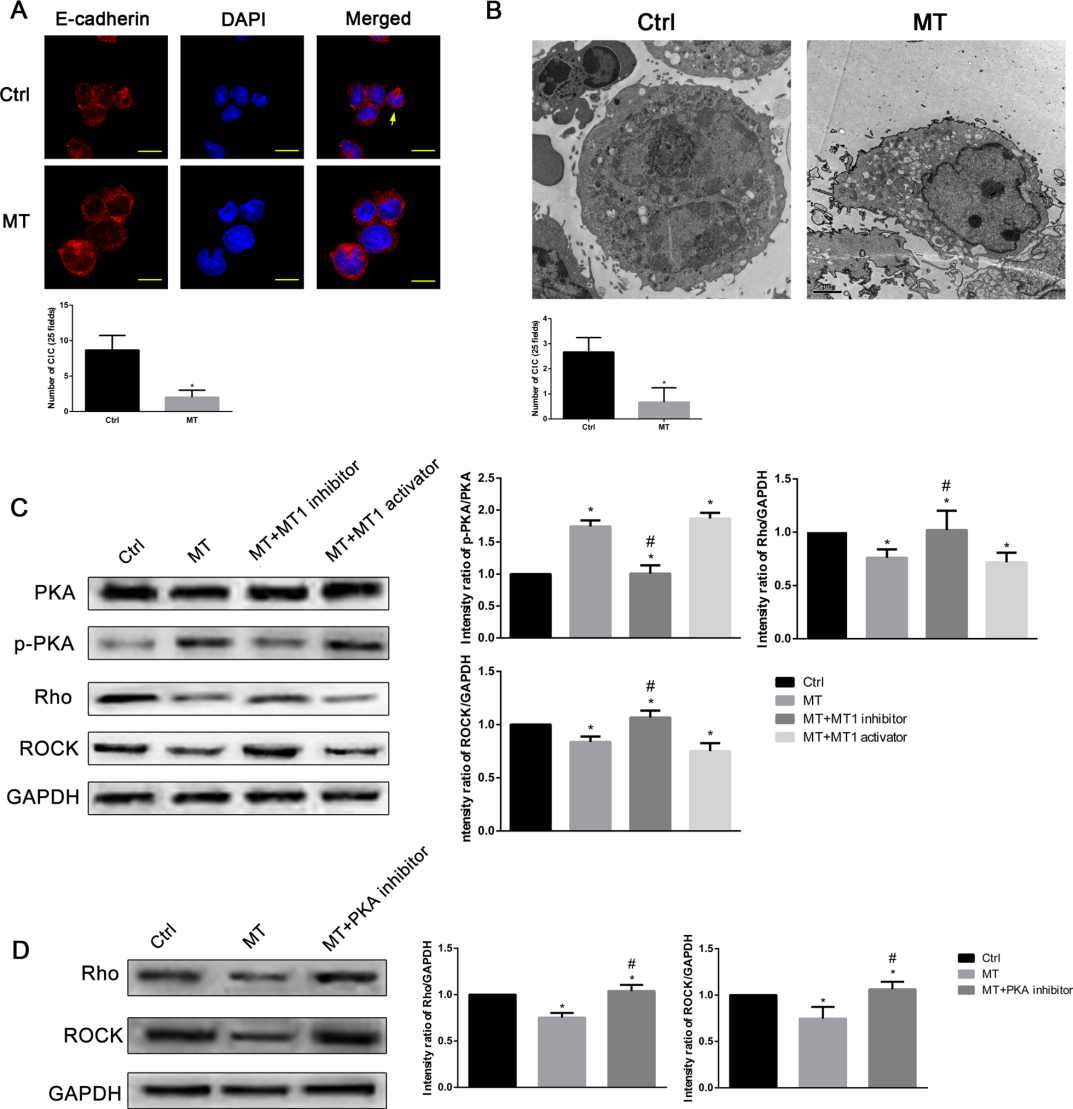
Effects of melatonin on CIC and OS cells in vitro. **A** Immunofluorescence of CIC in U2OS cells with or without melatonin treatment. *P < 0.05. n = 30 cells from three independent experiments. Yellow arrow indicated the CIC structure. Scale bar = 10 μm. **B** Electron microscope observed the CIC in U2OS cells with or without melatonin treatment. **C** Western blot shows PKA, p-PKA, Rho, ROCK expression in U2OS cells treated with melatonin, melatonin + MT1 inhibitor and melatonin + MT1 activator. **D** Western blot shows Rho and ROCK expressions in U2OS cells treated with melatonin, and melatonin + PKA inhibitor. *P < 0.05 vs. control, ^#^P < 0.05 vs. MT. Data are expressed as the mean ± SD of three independent experiments relative to control

In order to investigate the effect of melatonin on CIC phenomenon, U2OS cells were treated with melatonin and CIC were determined by immunofluorescence analysis and electron microscope. As shown in Fig. 4A and B, the number of CIC structure was significantly reduced with melatonin treatment. It is reported that ROCK proteins positively regulate CIC formation [38]. In order to investigate the molecular mechanism of melatonin on CIC formation, we treated the U2OS cells with melatonin (MT), MT + MT1 activator (7-Desmethyl-agomelatine) or MT + MT1 inhibitor (S26131) and evaluated the levels of PKA and Rho/ROCK. We found that both MT and MT + MT1 activator could promote PKA phosphorylation, and reduced Rho and ROCK expressions. In contrast, MT + MT1 inhibitor reversed the above results (Fig. 4C). We also found that MT + PKA inhibitor reversed the inhibitory effects of MT on Rho and ROCK expressions (Fig. 4D). These results indicated that melatonin may play an important role in inhibiting the Rho/ROCK signaling pathway of CIC by activating the PKA-Rho/ROCK signaling pathway, thus inhibiting CIC.

### Effect of melatonin on mitochondrial physiology of OS cells

**Fig. 5:**
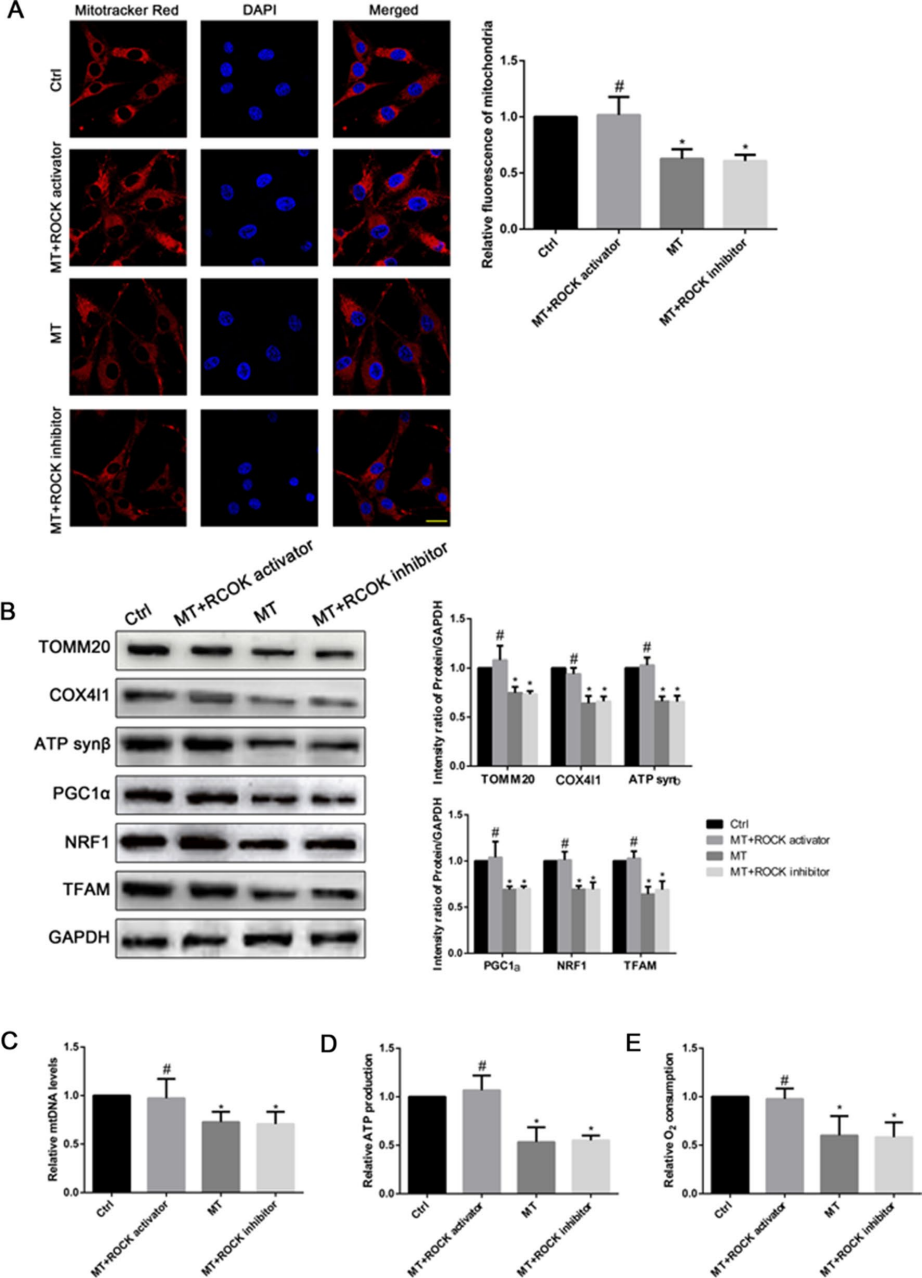
Effects of CIC on mitochondrial physiology of OS cells. U2OS cells were treated with melatonin, melatonin + ROCK activator and melatonin + ROCK inhibitor for 24 h. **A** Immunofluorescence of mitochondria in U2OS cells. Scale bar = 20 μm. **B** Western blot shows TOM20, COX4I1, ATP synβ, PGC1α, NRF1 and TFAM expressions. **C** mtDNA levels of U2OS cells. **D** Mitochondrial ATP production in U2OS cells. **E** Mitochondrial O_2_ consumption in U2OS cells. *P < 0.05 vs. control, ^#^P < 0.05 vs. MT. Data are expressed as mean ± SD fold induction of three independent experiments relative to control

CIC formation between tumor cells has been reported to alter energy metabolism, which closely related to the occurrence and development of tumors [31, 32]. We treated the U2OS cells with MT, MT + ROCK activator (pentanoic acid) [39], or MT + ROCK inhibitor (ROCK inhibitor-2) [40], and determined the relative fluorescence of mitochondria by immunofluorescence. We observed that MT and MT + ROCK inhibitor reduced the relative fluorescence of mitochondria in OS cells (P < 0.05, Fig. 5A), downregulated the expression of mitochondrial biogenesis-related proteins PGC1α, NRF1 and TFAM, and reduced the expression of mitochondrial biomarkers TOM20, COX-4l1 and ATP Synβ (P < 0.05, Fig. 5B). In contrast, MT + ROCK activator reversed the above results. We also found significantly higher mtDNA levels in the control and the MT + ROCK activator groups than in the MT and MT + ROCK inhibitor groups (P < 0.05, Fig. 5C). ATP production and O2 consumption were found to be higher in the control and the MT + ROCK inhibitor groups than in the MT and MT + ROCK inhibitor groups (Fig. 5D and E). These findings suggested that melatonin inhibited mitochondrial biogenesis and function in OS cells through the Rho/ROCK-mediated CIC pathway.

### Pharmacokinetics and in vivo safety of melatonin

**Fig. 6:**
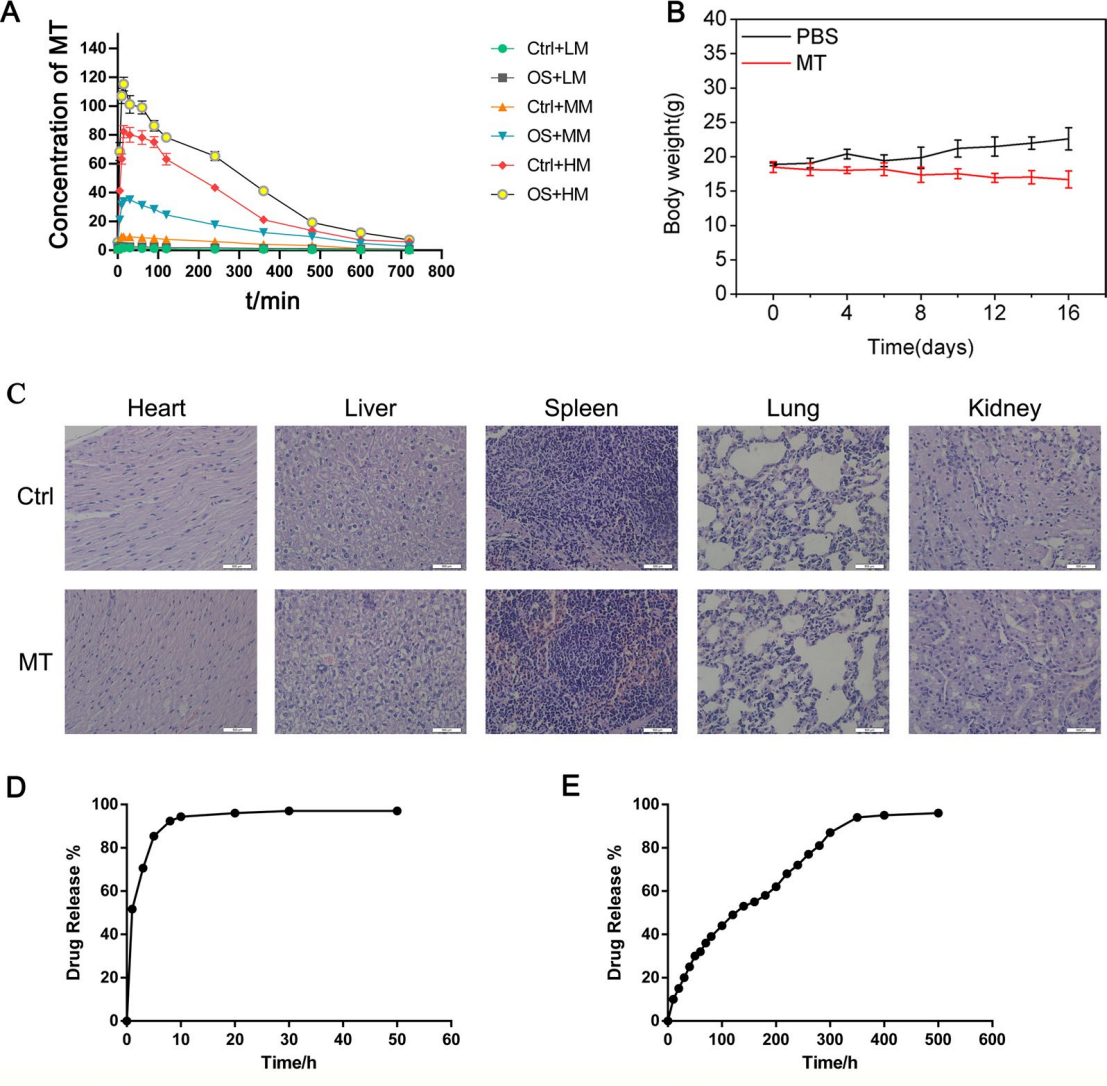
Pharmacokinetics and in vivo safety of melatonin. **A** Concentration of melatonin at different time points of mice with or without tumor. **B** Changes of body weight. **C** Ex vivo histological analyses of main organ sections from U2OS osteosarcoma-xenografted mice after melatonin treatment. **D** The drug release rate of melatonin. **E** The drug release rate of melatonin loaded SunP Gel G1

The pharmacokinetics of melatonin in mice were studied by injecting melatonin into the tail vein at 10, 100 and 1000 mg/kg according to body weight, and collecting blood from the fundus vein to observe the changes of melatonin concentration in the venous blood. The drug concentrations at different time points are shown in Fig. 6A and Table 2. Melatonin concentration peaked at 30 min after administration maintained with slight decrease for 6 h, and gradually decreased thereafter to near baseline levels at 12 h.

**Table 2:** The concentration of melatonin in the plasma of mice

t/min	LM			MM			HM		
	Ctrl	OS	P	Ctrl	OS	P	Ctrl	OS	P
0	0.514 ± 0.075	1.024 ± 0.112	< 0.05	0.671 ± 0.815	2.631 ± 0.916	< 0.05	3.751 ± 0.975	5.543 ± 0.893	< 0.05
5	0.853 ± 0.115	1.873 ± 0.303	< 0.05	5.034 ± 1.326	21.026 ± 2.116	< 0.05	41.267 ± 1.715	68.375 ± 6.326	< 0.05
10	1.602 ± 0.157	2.724 ± 0.312	< 0.05	9.423 ± 1.723	31.317 ± 1.893	< 0.05	63.365 ± 3.612	107.067 ± 5.215	< 0.05
15	1.531 ± 0.142	2.513 ± 0.287	< 0.05	9.324 ± 1.825	33.861 ± 2.004	< 0.05	82.326 ± 4.267	115.335 ± 4.756	< 0.05
30	1.501 ± 0.145	2.471 ± 0.275	< 0.05	9.253 ± 1.741	35.034 ± 1.246	< 0.05	80.034 ± 5.109	101.231 ± 6.216	< 0.05
60	1.234 ± 0.092	2.364 ± 0.259	< 0.05	8.732 ± 1.626	31.125 ± 0.896	< 0.05	78.131 ± 4.756	99.034 ± 4.489	< 0.05
90	1.174 ± 0.081	2.157 ± 0.221	< 0.05	8.328 ± 1.273	28.471 ± 0.791	< 0.05	75.022 ± 3.792	86.328 ± 3.681	< 0.05
120	0.911 ± 0.067	1.873 ± 0.203	< 0.05	7.519 ± 0.935	24.532 ± 0.687	< 0.05	63.237 ± 4.102	78.265 ± 2.436	< 0.05
240	0.734 ± 0.045	1.684 ± 0.197	< 0.05	6.051 ± 0.587	17.672 ± 0.836	< 0.05	43.414 ± 2.891	65.354 ± 3.196	< 0.05
360	0.551 ± 0.031	1.341 ± 0.164	< 0.05	4.034 ± 0.435	12.336 ± 0.578	< 0.05	21.243 ± 1.946	41.246 ± 2.262	< 0.05
480	0.374 ± 0.025	1.216 ± 0.137	< 0.05	3.276 ± 0.375	9.477 ± 0.433	< 0.05	13.635 ± 2.121	19.348 ± 1.757	< 0.05
600	0.238 ± 0.023	0.875 ± 0.125	< 0.05	1.216 ± 0.203	4.827 ± 0.327	< 0.05	7.044 ± 1.128	12.134 ± 1.113	< 0.05
720	0.124 ± 0.017	0.649 ± 0.116	< 0.05	0.671 ± 0.194	2.715 ± 0.366	< 0.05	5.768 ± 1.142	7.215 ± 0.575	< 0.05

Statistical significance between the two groups was analyzed by the Student’s t-test

*LM* low concentration of melatonin, *MM* medium concentration of melatonin, *HM* high concentration of melatonin, *OS* osteosarcoma

The safety of melatonin in mice was further studied by injecting melatonin into the tail vein of mice once every 2 days at 3 mg each time. After 30 days, the heart, liver, spleen, lung, kidney and other important organs were collected, sectioned and stained with HE for microscopy observation. The weight of all mice was stable without notable changes (Fig. 6B) and no organ damage was observed in mice treated with melatonin (Fig. 6C). The LE and LC of melatonin loaded SunP Gel G1 at mass concentration of 5 g/l are shown in Table 3. Along with the increasing of the mass ratio of melatonin and SunP Gel G1, the LE and LC were increased. The LE reached peak at mass ratio of 1:2 and was decreased at mass ratio of 2:1. The mass ratio of 1:2 was selected for further experiments.

**Table 3:** The loading efficiency and capacity of melatonin-loaded SunP Gel G1 gel

	m (MT): m (SunP Gel G1)				
	1: 4	1: 3	1: 2	1: 1	2: 1
LE (%)	67.85	76.22	85.38	79.72	77.55
LC (%)	13.57	19.06	28.46	39.86	51.70

*LE* loading efficiency, *LC* loading capacity, *m* mass

The drug release rates of melatonin and melatonin loaded SunP Gel G1 are shown in Fig. 6D and E. The cumulative release rate of melatonin has reached 96.3% at 10 h, while the drug release cycle of melatonin loaded SunP Gel G1 can reach 14 days and there is no obvious burst release. These results suggest melatonin loaded SunP Gel G1 has ability to control the release of melatonin and effectively achieves the sustained release of melatonin.

### Effect of 3D-printed Mg–PCL–MT scaffold on OS progression in vivo

**Fig. 7:**
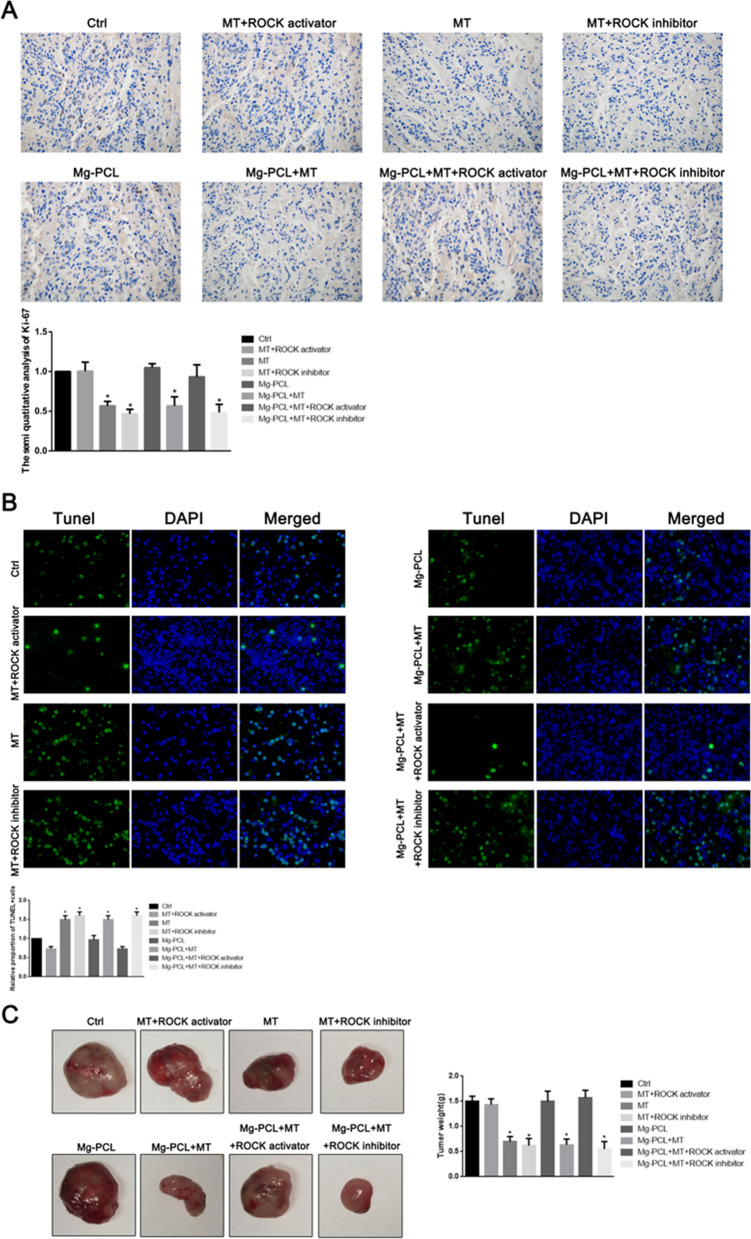
Effect of 3D-printed Mg–PCL-MT scaffold on OS in vivo. **A** immunofluorescence and quantitative analysis of Ki-67 in MT + ROCK activator, MT, MT + ROCK inhibitor, Mg–PCL, Mg–PCL + MT, Mg–PCL + MT + ROCK activator and Mg–PCL + MT + ROCK inhibitor groups. *P < 0.05 vs. control. **B** TUNEL staining of tumor and quantitative analysis in MT + ROCK activator, MT, MT + ROCK inhibitor, Mg–PCL, Mg–PCL + MT, Mg–PCL + MT + ROCK activator and Mg–PCL + MT + ROCK inhibitor groups. *P < 0.05 vs. control. **C** Represent photographs and tumor weight curves of U2OS tumors in MT + ROCK activator, MT, MT + ROCK inhibitor, Mg–PCL, Mg–PCL + MT, Mg–PCL + MT + ROCK activator and Mg–PCL + MT + ROCK inhibitor groups. Values are presented as the mean ± SD of three independent experiments. *P < 0.05 vs. control

Since the Mg–PCL scaffold could promote the bone growth and melatonin could inhibit CIC phenomenon, we speculated that melatonin-loaded Mg–PCL scaffold might inhibit OS progression. We found that MT and MT + ROCK inhibitor effectively inhibited the cell proliferation (Fig. 7A) and tumor weight (Fig. 7C) and promoted cell apoptosis (Fig. 7B) of xenograft tumors in nude mice with implanted U2OS cells (P < 0.05), while MT + ROCK activator had no significant effect (P > 0.05). These results demonstrated that melatonin inhibited OS growth in vivo and this inhibition could be abolished by ROCK activator. In addition, Mg–PCL + MT, Mg–PCL + MT + ROCK activator and Mg–PCL + MT + ROCK inhibitor demonstrated similar effect on cell proliferation with those without Mg–PCL.

**Fig. 8:**
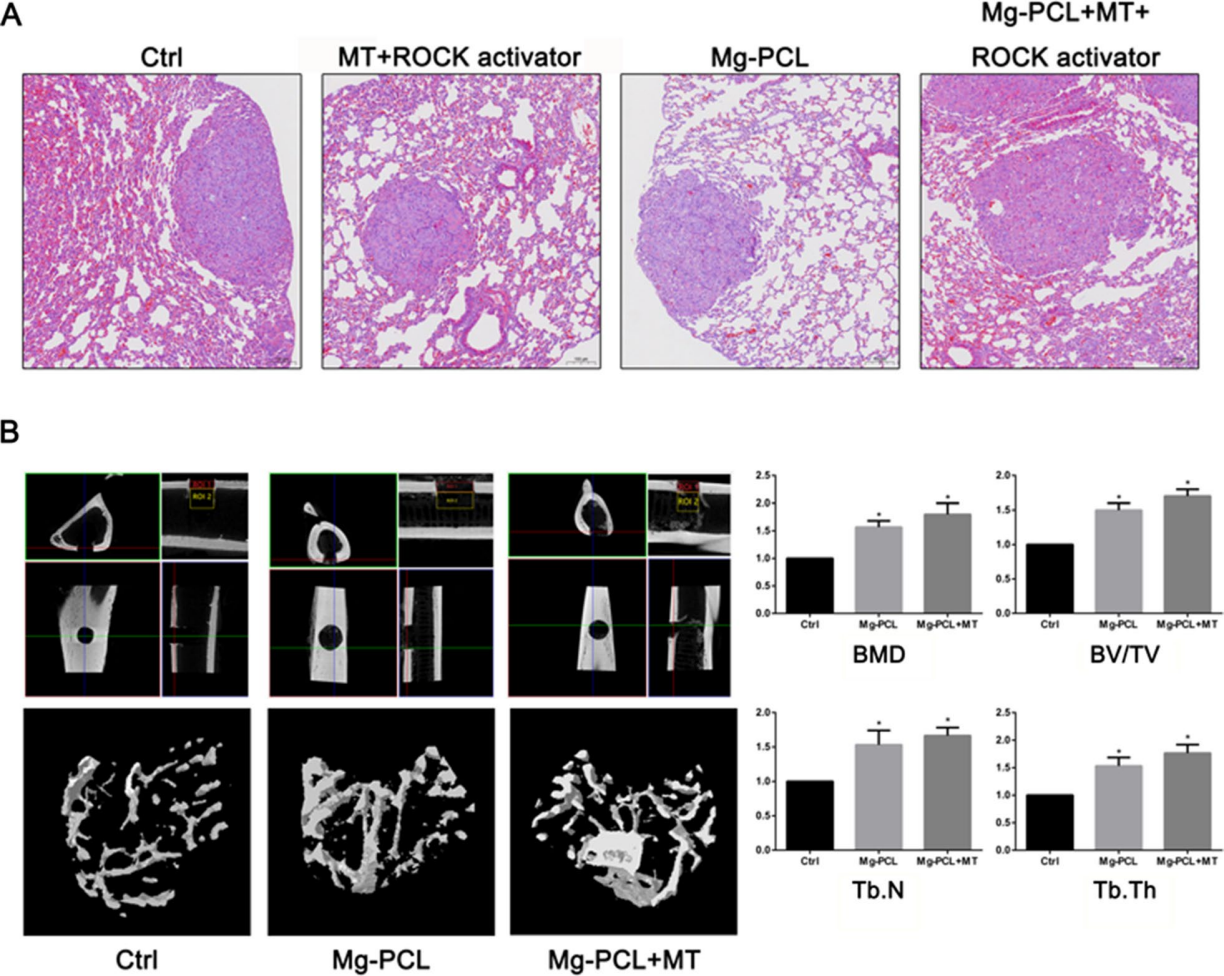
Effect of 3D-printed Mg–PCL–MT scaffold on the bone growth in vivo. **A** HE staining of tumor sections. **B** MicroCT scan of Control, Mg–PCL and Mg–PCL + MT. BMD, BV/TV, Tb.N and Tb.Th were determined and compared. The arrows indicated the newly formed callus. *P < 0.05 vs. control

HE staining demonstrated that lung metastasis occurred in the control, MT + ROCK activator, Mg–PCL scaffold and Mg–PCL + MT + ROCK activator groups, while no organ metastasis was observed in other groups (Fig. 8A). We established an in vivo OS model and inserted the Mg–PCL implant and Mg–PCL-MT implant group into the front edge of the tibial plateau. MicroCT scan results of mice tibia showed that the formation of callus in the Mg–PCL implant group and Mg–PCL + MT implant group was increased and the static bone microstructure index was the highest in Mg–PCL + MT implant group (Fig. 8B).

### Effect of 3D-printed Mg–PCL–MT scaffold on OS cell invasion and migration in vitro

**Fig. 9:**
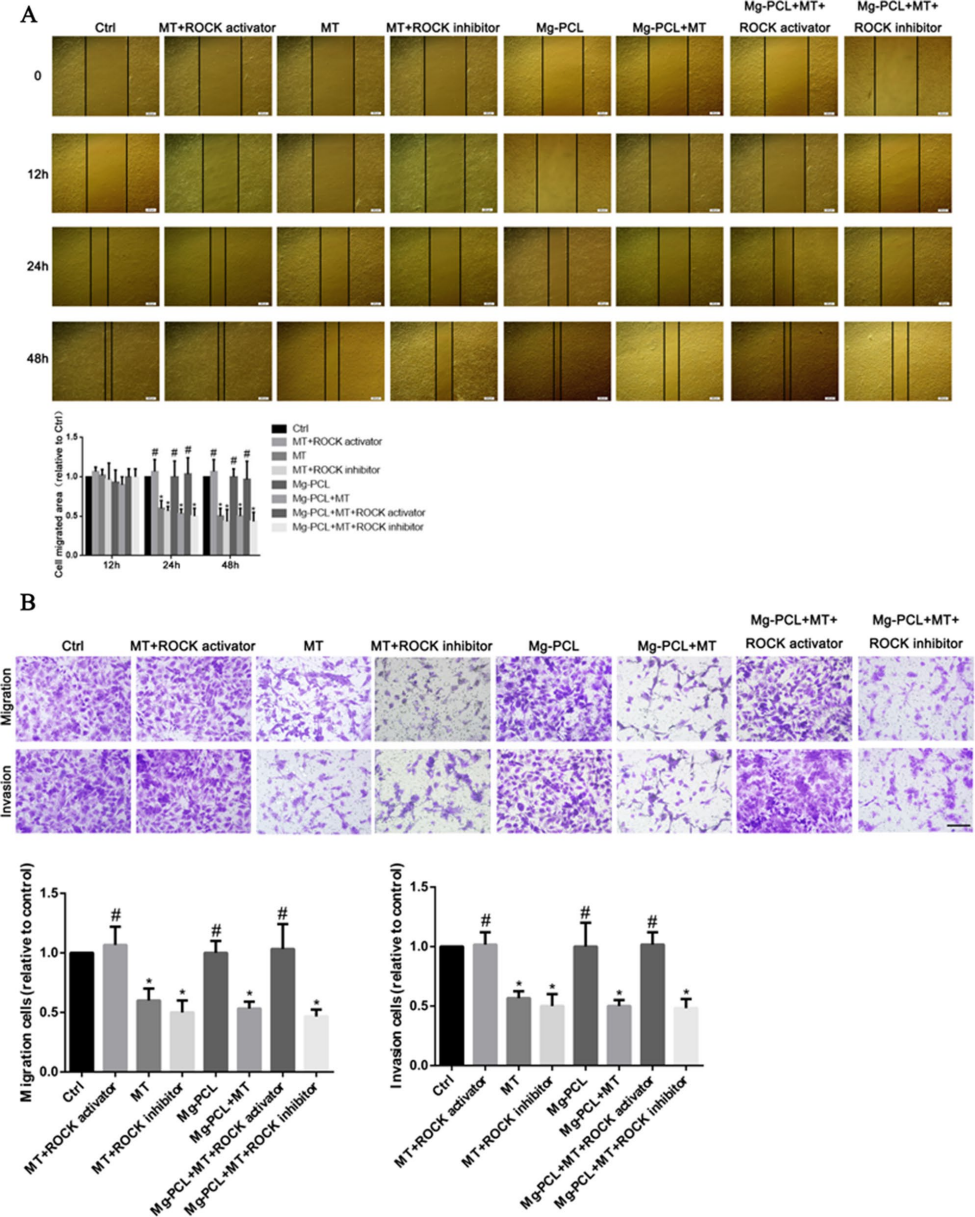
Effect of 3D-printed Mg–PCL–MT scaffold on OS cell invasion and migration in vitro. **A** Wound healing results and quantitative analysis of U2OS cells under MT + ROCK activator, MT, and MT + ROCK inhibitor, Mg–PCL, Mg–PCL + MT, Mg–PCL + MT + ROCK activator and Mg–PCL + MT + ROCK inhibitor treatments at 0, 12 h, 24 and 48 h. Scale bar = 200 μm. **B** Transwell assay of the U2OS cells under MT + ROCK activator, MT, and MT + ROCK inhibitor, Mg–PCL, Mg–PCL + MT, Mg–PCL + MT + ROCK activator and Mg–PCL + MT + ROCK inhibitor treatments for 24 h. Scale bar = 50 μm. Values represent the mean ± SD of three independent experiments. *P < 0.05 vs. control

The effect of 3D-printed Mg–PCL–MT scaffold on OS cell invasion and migration were assessed by wound-healing assay and Transwell. The wound healing demonstrated that the scratch width was wider in the MT + ROCK inhibitor and the MT groups, but narrower in the MT + ROCK activator group (Fig. 9A). Quantitative analysis suggested that MT and MT + ROCK inhibitor groups significantly decreased cell migrated area relative to control (P < 0.05), while MT + ROCK activator not (P > 0.05). In addition, Mg–PCL + MT, Mg–PCL + MT + ROCK activator and Mg–PCL + MT + ROCK inhibitor demonstrated similar effect on cell migration with those without Mg–PCL (Fig. 9A). Transwell results showed that the number of invaded and migrated cells in the MT and the MT + ROCK inhibitor groups was significantly lower than that in the MT + ROCK activator group and the control groups (Fig. 9B). Mg–PCL + MT, Mg–PCL + MT + ROCK activator and Mg–PCL + MT + ROCK inhibitor demonstrated similar effect on cell migration and invasion with those without Mg–PCL. These results suggested that 3D-printed Mg–PCL–MT scaffold showed similar effects on inhibiting the invasion and migration of OS cells with melatonin.

## Discussion

At present, clinical treatment of OS commonly includes preoperative/neoadjuvant chemotherapy, surgical resection of primary and metastatic tumor areas, and postoperative chemotherapy [41]. Bone defects derived from surgical resection need to be reconstructed using biomaterials. PCL is a biocompatible and biodegradable polymer, however, its biological activity is poor [42]. It is reported that magnesium ion is essential for new bone formation and could induce increase in osteogenic activity [9, 10]. By 3D printing technology, we developed an Mg–PCL scaffold and assessed its effects and molecular mechanism on inhibiting OS growth and metastasis. Results demonstrated that Mg–PCL scaffold could promote bone growth and might be an ideal candidate for bone tissue engineering; however, it can’t inhibit tumor metastasis. This result is partly consistent with the study of Zhao et al., which demonstrated PCL/10% Mg composite scaffolds could promote bone defect repair at an early stage with good cytocompatibility [43].

OS cells are not sensitive to radiotherapy [44, 45], and the existing chemotherapeutic anti-tumor drugs are limited by poor tumor specificity which often results in systemic toxicity. In addition, long-term use of chemotherapy drugs can lead to drug resistance as well as decrease in life quality of patients [46, 47]. Melatonin, as a hormone naturally produced in the human body, has the natural advantages of good biocompatibility and high safety. In this study, the safety of melatonin was proven by HE staining and pharmacokinetics analyses in the liver, lung and kidney of mice. However, the half-life of melatonin is short, which hampers its clinical use in medical materials. Drug delivery system was investigated to settle this problem. For example, Altindal et al. [48] prepared a sustained melatonin release system to inhibit OS proliferation. In this study, we used hydrogel to load melatonin on Mg–PCL scaffold. The hydrogel can construct a good biologically active interface on the surface of the Mg–PCL scaffold, and has better biocompatibility [49]. Our results suggested the cumulative release rate of melatonin has reached 96.3% at 10 h, while the drug release cycle of melatonin loaded SunP Gel G1 can reach 14 days and there is no obvious burst release. These results suggested that the hydrogel can slow down the release of melatonin to achieve the effect of sustained release. In addition, through in vivo and in vitro experiments, the exact therapeutic effects of melatonin on OS were confirmed. These results demonstrated that melatonin is an effective and valuable treatment option for OS. In the result of MicroCT scan, it is showed that the formation of callus in the Mg–PCL implant group and Mg–PCL + MT implant group was increased and the static bone microstructure index was the highest in Mg–PCL + MT implant group. There have been several studies on the treatment of OS with melatonin. Qu et al. [2] and Liu et al. [50] showed that melatonin inhibited the proliferation and migration of OS cells. Maria et al. and Balci Yuce et al. demonstrated that melatonin could regulate bone formation, growth and differentiation as well as exert anti-osteoporosis effects [23, 24]. Zhang et al. demonstrated that melatonin may reduce the level of autophagy in osteoblasts and delay diabetes-induced osteoporosis by inhibiting the ERK signaling pathway [51]. However, the underlying mechanism via which melatonin inhibits OS progression has not been systematically studied or elucidated.

Unlike conventional chemotherapeutic drugs which act by poisoning tumor cells or activating tumor immunity, treatment targeted towards the CIC phenomenon in OS tissues would suppress the highly energy-consuming nature of OS. As a highly specific treatment, this would have no side effects on normal tissue cells and would effectively inhibit OS development while exerting the lowest cost in terms of tissue damage. In this study, CICs were found in clinical samples of OS patients. With the development of research on the CIC phenomenon, its biological significance is gradually becoming clear. Overholtzer et al. has summarized the possible biological roles of CIC [29], and described that they largely depended on the types of interacting cells. Therefore, we further studied the effects of melatonin on the CIC phenomenon, and elucidated the molecular biological mechanisms connecting melatonin and OS treatment.

The relationship between melatonin and CIC has not been reported, but the relationship between Rho/ROCK and CIC formation has been identified [38]. The effect of melatonin on cAMP/PKA -Rho/ROCK signaling pathway were determined. We found MT1 activator could activate the cAMP/PKA signaling pathway, which was consistent with the report of Schuster et al. [52], and also confirmed a regulatory relationship between PKA and Rho [53]. Therefore, we proposed that melatonin can activate the cAMP/PKA pathway through its receptor, and consequently inhibit the Rho/ROCK pathway, thus interfering with the formation of CIC. Our hypothesis was confirmed, and we successfully identified the regulatory relationship between melatonin and CIC for the first time.

CIC formation between tumor cells has been reported to alter energy metabolism, which closely related to the occurrence and development of tumors [31, 32]. Mitochondria are organelles that carry genetic information and energy. Wang et al. demonstrated that mitochondria could be transferred among cells through CIC phenomenon [54]. We therefore confirm that CIC affects OS cell function by interfering with its energy metabolism. Previous studies have revealed that under extreme conditions such as a nutrient-deficient microenvironment, host cells adapted by swallowing effector cells. Leibman et al. after observing the degradation of lymphocytes in host oocytes, suggested that degraded lymphocytes may provide nutrition for host cells [55]. Trowell et al. proposed a similar hypothesis, and observed lymphocyte degradation in fibroblasts and thymic reticulum cells in vitro [56]. Ioachim have demonstrated lymphocyte degradation in a variety of cells in vitro [29, 57], while Fais et al. found that metastatic melanoma cells could survive by ingesting live T cells in the absence of nutrients [31, 32, 58]. In this study, we showed that melatonin inhibited mitochondrial biogenesis and function in OS cells through the Rho/ROCK-mediated CIC pathway.

In the present study, we found that MT1 expression was upregulated in OS cells. We demonstrate that Mg–PCL–MT could benefit the tumor suppression of melatonin, and our novel melatonin-loaded Mg–PCL scaffolds synergistically inhibited the key CIC pathway, Rho/ROCK, through the cAMP/PKA signaling pathway, interfering with the mitochondrial physiology of OS cells, and thus exerted an anti-invasion and anti-metastasis effect on OS cells. Our results, for the first time, lay foundation for developing melatonin-loaded Mg–PCL scaffolds into a new local adjuvant therapy for OS. Despite having systematically evaluated the molecular biological mechanisms of melatonin in the CIC phenomenon and in OS progression, the underlying mechanism of MT1 expression in OS cells, as well as the epigenetics and proteomics of patient samples, needs to be further explored.

## Data Availability

The data that support the findings of this study are available on request from the corresponding author.
